# Directed Discovery of Tetrapeptide Emulsifiers

**DOI:** 10.3389/fchem.2022.822868

**Published:** 2022-02-17

**Authors:** Gary G. Scott, Tim Börner, Martin E. Leser, Tim J. Wooster, Tell Tuttle

**Affiliations:** ^1^ Department of Pure and Applied Chemistry, University of Strathclyde, Glasgow, United Kingdom; ^2^ Institute of Materials Sciences, Nestlé Research Center, Lausanne, Switzerland

**Keywords:** peptide, emulsifier, coarse grain, modelling, simulation

## Abstract

Oil in water emulsions are an important class of soft material that are used in the food, cosmetic, and biomedical industries. These materials are formed through the use of emulsifiers that are able to stabilize oil droplets in water. Historically emulsifiers have been developed from lipids or from large biomolecules such as proteins. However, the ability to use short peptides, which have favorable degradability and toxicity profiles is seen as an attractive alternative. In this work, we demonstrate that it is possible to design emulsifiers from short (tetra) peptides that have tunability (i.e., the surface activity of the emulsion can be tuned according to the peptide primary sequence). This design process is achieved by applying coarse grain molecular dynamics simulation to consecutively reduce the molecular search space from the 83,521 candidates initially considered in the screen to four top ranking candidates that were then studied experimentally. The results of the experimental study correspond well to the predicted results from the computational screening verifying the potential of this screening methodology to be applied to a range of different molecular systems.

## Introduction

Over the past few years, there has been an emergence of the use of peptides for the development of new materials for use in the food, cosmetic and biomedical industries.(Zelzer and Ulijn, 2010; Zhao et al., 2010; Bai et al., 2014; Frederix et al., 2015; Scott et al., 2016; Abul-Haija et al., 2017; Lampel et al., 2017; Zhu et al., 2018). Peptides provide a cheap and biocompatible building block for creating new materials with tunable properties. The discovery of Fmoc as a capping group for promoting peptide self-assembly, showed that the structure of the materials could be varied through the peptide sequence, (Fleming and Ulijn, 2014), however, the presence of large aromatic groups such as Fmoc can be harmful (Ferguson and Denny, 2007). Development of new peptide materials without the presence of large aromatic capping groups showed that similar materials could be formed with similar tunable properties (Frederix et al., 2015). Furthermore, these materials can be utilized in multi-solvent systems to created stable emulsions (Scott et al., 2016; Aviño et al., 2017; Castelletto et al., 2019; García-Moreno et al., 2020; Wychowaniec et al., 2020).

Previous examples of short peptide based emulsifiers have relied on longer peptides (9–26 amino acids in length) that have a classical surfactant like structure (Castelletto et al., 2019) or alternatively form identifiable structural motifs that are present in naturally occurring emulsifiers (García-Moreno et al., 2020). In the case where shorter (di-tetrapeptides) have been identified, or designed as emulsifiers a recurring theme is the presence of aromatic amino acids such as phenylalanine and tyrosine (Scott et al., 2016; Aviño et al., 2017; Wychowaniec et al., 2020). In this work a new approach for the discovery of peptides emulsifiers that are able to break these design rules is explored.

Since the discovery by Reches and Gazit of the ability of diphenylalanine (FF) to self-assemble, (Reches and Gazit, 2003), peptides have been increasingly investigated for their ability to structure materials. Of particular interest for the food and cosmetics industries is the GRAS (Generally Regarded As Safe) status of amino acids (Smriga, 2020). However, the use of aromatic amino acids such as phenylalanine (F), tryptophan (W), and tyrosine (Y) is considered less desirable due to the propensity of these amino acids to form very stable supramolecular structures that can withstand metabolization.

The recent research into the ability of short peptides to act as structural agents in soft materials has further emphasized the role of aromatic amino acids for forming nanofibrous networks that are capable of stabilizing soft materials, with a review of the literature highlighting the importance of an aromatic dyad in forming peptide-based materials (Lampel et al., 2018). Based on the industrial desire to move away from this motif, we explored the possibility of stabilizing emulsions using short peptides (tetrapeptides) formed exclusively from the gene-encoded amino acids, excluding F, W, and Y as the use of short-peptide containing aromatic amino acids that are able to act as emulsifiers through self-assembly has been studied and protected elsewhere (Ulijn et al., 2017).

The exclusion of F, W, and Y, result in a potential 83,521 unique peptide sequences. This number of potential candidates is not feasible to screen experimentally and even applying the coarse-grain screening process that have been developed in our lab would be prohibitively time-consuming (*ca.* 750,000 cpu hours) for a complete screen of this size. Therefore, this work firstly describes the filtering process that was applied to limit the candidates that were selected for screening. The second stage of the work involves the analysis of the top-scoring candidates before the experimental evaluation of the selected candidates is discussed.

## Materials and Methods

**FIGURE 1:**
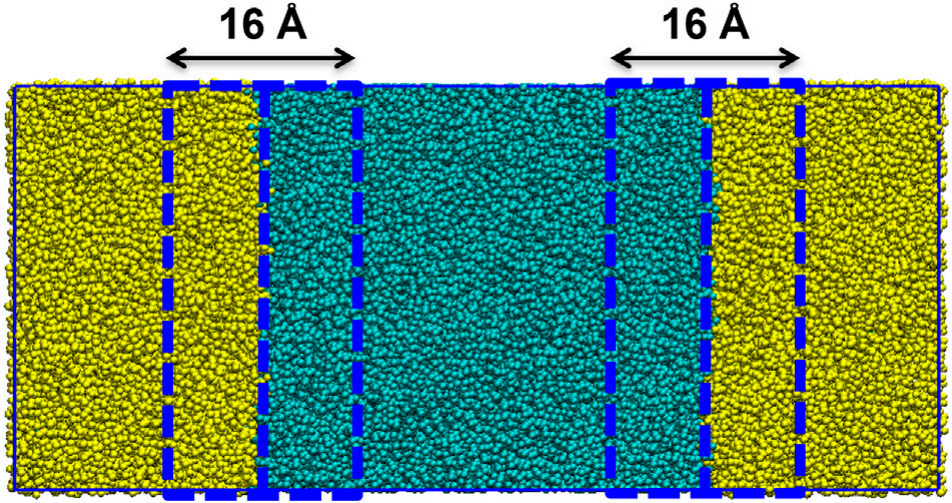
Construction of the solvent box (25 nm × 12.5 × 12.5 nm) with the aqueous phase (turquois beads) centered between the octane phase (yellow beads). The interface between the phases is defined as within 8 Å of the octane water boundary.

Each system is setup using GROMACS version 4.5.3 (Hess et al., 2008). Using GROMACS commands, a box with dimensions 25 × 12.5 × 12.5 nm was created. To the box, 600 molecules of the designated peptide sequence, in the zwitterionic form, and were added. This box was then solvated with a pre-equilibrated box containing water and octane. The box was constructed such that the aqueous phase was in the center of the box, thereby creating two octane water interfaces (Figure 1). Na^+^ or Cl^−^ ions were added to neutralise all charges. Each system has periodic boundary conditions in operation.

The Martini force field (Marrink et al., 2007) was used to model the system as this has previously been demonstrated to provide an accurate representation of the emulsifiers (Marrink and Tieleman, 2013; Scott et al., 2016; Sun et al., 2017; Wijaya and Hertadi, 2019; Crespo et al., 2020). Each system is then minimised for a maximum of 5,000 steps to relax constrained overlaps and bonds. This is followed by an equilibration and production run for 100 ns real time (25 ns simulations time) within the NVT ensemble. The simulations were carried out in GROMACS v5 (Abraham et al., 2015).

The octane water interface was employed as the Martini Forcefield has been well-parameterized to reproduce the partitioning behavior at this interface. While the experimental comparison focuses on the water-air interface, this direct comparison is not reliable within the forcefield as used. However, the goal of this work is to describe a method that allows the rapid identification of potential candidates from a very large sequence space for further investigation, rather than an explicit comparison between computational, and experimental results. As such, the method described provides an acceptable trade-off between the accuracy and efficiency of the computational method and the ability to target the desired experimental property.

### Measurement of the dynamic surface tension

The water-air dynamic surface tension, γ(t), was measured using a pendant drop and bubble tensiometer (also denoted as Profile Analysis tensiometer) from Teclis, France (TRACKER tensiometer). Drop and bubble profile tensiometers have been developed mainly for the measurement of surface tensions from the shape of drops or bubbles. These shapes are caused by the balance of gravity and interfacial tension described by the Gauss-Laplace equation (Leser et al., 2005). In the present study a bubble was dipped into a glass cuvette containing the peptide solution using a U-shaped metal capillary. The bubble volume was 10 µl. The surface tension between air and pure demineralized water is 72 mN/m at a temperature of 25°C; Experiments were performed in the Area control mode; The reproducibility of the γ(t) curves was within 0.5 and 1 mN/m for the same ageing time (30 min).

The tetrapeptides were synthetized and provided in Lyophilized form by ProteoGenix, France. They are supplied in their acetate form (buffer). The lyophilized powders were stored at −20°C before usage. Their chemical purity is better than 96%.

## Results and Discussion

In order to determine whether a peptide had the ability to act as an emulsifier we considered the tendency of the peptide to adsorb at the water/octane interface as a key parameter to evaluate in the screening process. The % adsorbance (%ADS) of a peptide sequence was evaluated as the ratio between the total number of peptides that were at an interface at the completion of the screening simulation and the total number of peptides in system. A peptide is considered to be at the interface if any bead of the peptide is within 8 Å of the octane-water boundary (Figure 1).

**FIGURE 2:**
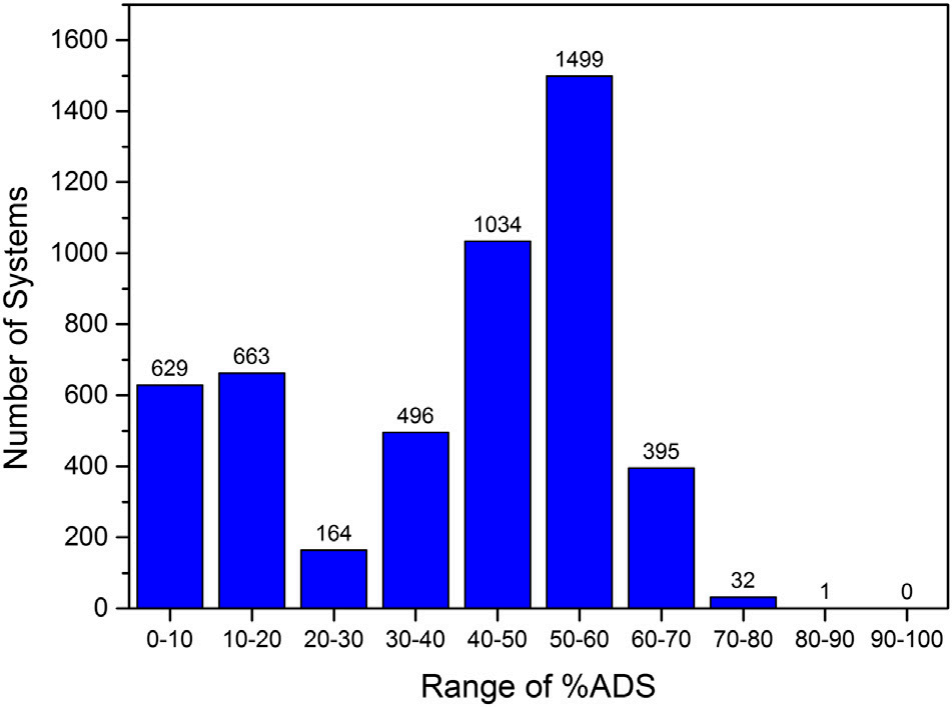
Range of %ADS values calculated for the 4,913 tripeptides that do not contain F, W, and Y.

Initially, the 4,913 tripeptides that do not contain F, W, and Y were screened to evaluate whether particular peptide sequences gave rise to higher adsorption abilities. This number of peptides can be screened relatively efficiently and potential patterns that arise in the positioning of amino acids in the peptide sequence to enhance the interfacial properties can be used to derive a subset of tetrapeptides to screen. In order to determine whether there was a significant sequence dependence on the adsorption ability of the peptide, the range of %ADS calculated for the tripeptide screen was investigated (Figure 2).

Figure 2 shows that the tripeptides demonstrate a wide range of interfacial behavior. Approximately 30% of the tripeptides were found to have very low interfacial activity with less than 30% of the number of peptides in the simulation accumulating at the interface by the completion of the 100 ns simulation. In contrast, 1,927 sequences (*ca.* 40%) showed a %ADS of >50% with 32 of these sequences demonstrating high interfacial activity (>70%, Figure 2). The %ADS for each tripeptide sequence is provided in the Supplementary Material. However, the 32 top-scoring peptides do not present any obvious sequence patterns. Therefore, we carried out an analysis of the effect of the presence of an individual amino acid within the tripeptide sequence on the interfacial activity of the peptide.

**FIGURE 3:**
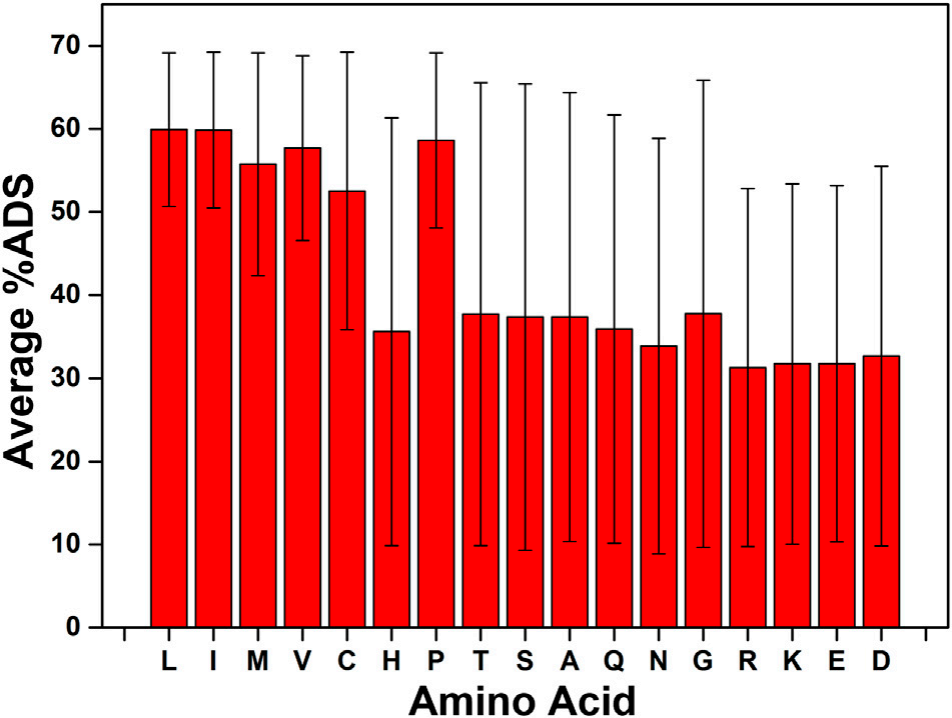
The ability for the presence of a single amino acid type to affect the %ADS of the tripeptide sequence. The average value is calculated for each amino acid as the average of the calculated %ADS for any tripeptide sequence that contains that amino acid. The error bars indicate the standard deviation (σ) across the population used to calculate the average.

To determine whether the presence of a specific amino acid in the tripeptide sequence had a strong influence on the interfacial activity of the peptide, the average %ADS for every tripeptide sequence containing a given amino acid was calculated, along with the standard deviation for the %ADS scores across the series of tripeptides containing the amino acid. This analysis reveals that, on average, the amino acids L, I, M, V, C, and P, lead to a greater tendency for the tripeptide to accumulate at the interface (average %ADS >50%, Figure 3). However, what is more striking than the average %ADS variation between the amino acids is the standard deviation among the %ADS values acquired for the population. The standard deviation indicates how deterministic the presence of the amino acid in the sequence is on the ability of the peptide to adsorb at the interface. The smallest standard deviations (σ) are observed for L (σ = 9.24) and I (σ = 9.37), which also have the highest average %ADS results (*ca.* 60%, Figure 3; see Supplementary Material for complete list of results).

Based on the analysis of the individual amino acids the inclusion of L or I in all potential tetrapeptide sequences was determined. In addition, a sequence preference analysis was carried out to determine if including the amino acid as the N/C-terminus or in the middle of the sequence was preferred. In the case of both L and I there is a slight preference for the termini positions (see Supplementary Material). The individual amino acid analysis also indicated that the presence of the charged amino acids (R, K, E, and D) had a detrimental effect on the interfacial activity of the tripeptide (the average %ADS for each of these amino acids is *ca.* 30%, Figure 3). Therefore, the charged amino acids were excluded from the set of tetrapeptides that were investigated for their ability to act as emulsifiers. The exclusion of R, K, E, and D further reduces the potential sequence space for tetrapeptides to 28,561.

As a result of the individual amino acid analysis and the identification of 32 tripeptides with high interfacial activity, the following rules were used to construct the screening set for tetrapeptides:

(1) Each of the tripeptides that had a %ADS >70% would be used as a starting motif with the remaining sequence positions filled by one of the 17 amino acids (*i.e.*, excluding F, W, and Y, but including D, E, K, and R). This leads to a total of 34 possible tetrapeptide sequences for each of the 32 tripeptide candidates as the additional amino acid can be positioned at either the C-terminus or the N-terminus.
(2) All possible tetrapeptide sequences that have *either* L *or* I in a terminal position, but that do not include F, W, Y, D, E, K, and or R.
(3) As a control set, we additionally created all tetrapeptides that include the di-arginine motif (RR), which was shown to be the worst performing (*i.e.*, produced the lowest average %ADS).

These rules led to a screening set of 11,722 tetrapeptides, including 1,026 from the control set (*ca.* 9% of the total number of sequences screened). The %ADS was calculated for all tetrapeptides included in the scan and ranged from 0.3–74.3%. To analyze the effect of the amino acid type within the tetrapeptide sequences we carried out a similar analysis as in the case of the tripeptides (Table 1). This analysis shows that, with the exception of D, E, K, and R, each amino acid has a similar contribution to the %ADS score, with both a consistent average %ADS (*ca.* 50%) and a lower σ relative to the tripeptide sequences (Table 1).

**TABLE 1:** Average contribution of each amino acid type to the %ADS of the tetrapeptides.

Amino acid	%ADS	σ	%ran^a^
A	51.9	9.5	23.9
G	51.6	9.6	22.8
I	51.5	5.6	48.8
M	51.1	7.5	18.4
P	51.1	5.9	19.8
C	51.1	7.8	24.3
L	50.9	5.6	56.9
V	50.5	6.7	19.1
Q	50.4	9.8	19.1
T	50.3	9.5	20.3
S	50.1	9.5	20.5
N	49.0	9.3	27.9
H	48.0	9.6	18.3
E	38.2	17.3	3.3
D	37.5	17.8	3.0
K	36.7	17.0	3.1
R	26.9	18.9	9.3

a Indicates the number of peptides that have been run that contain the amino acid indicated, as a percentage of the total number of tetrapeptides included in the screen (11,722).

The relative abundance of the amino acids in the screen (%ran, Table 1) are, predictably, dependent on the process by which the tetrapeptides were chosen. The amino acids I and L are present in *ca.* 50% of the tetrapeptide sequences as the majority of the sequences included within the screen were required to have either I or L as a terminal amino acid. Similarly, the charged amino acids (D, E, and K) are present in *ca.* 3% of the sequences as these amino acids were excluded from the main selection of tetrapeptide sequences (rule 2), the exception to this is the positively charged amino acid R, which is more prevalent in the population due to its use as a control motif in the screen (rule 3). As expected, the sequences that contain charged amino acids have a significantly lower average %ADS than the other amino acids, which suggests that the exclusion of these amino acids from the tetrapeptide screen, based on the results of the tripeptide screen is valid.

The lower %ADS of the charged amino acids is due to the higher solvation that these residues require. Charged residues interact with the water molecules to a greater extent than polar residues and therefore there is a closer association of the water to the peptide molecules. This disrupts the tight packing of the peptides at the interface and destabilises any emulsion droplets that would form. In addition, the standard deviation of the %ADS of charged tetrapeptides is relatively high indicating a large spread of %ADS values.

These observations are validated by the highest and lowest ranking of the tetrapeptide (Table 2). The majority of tetrapeptides that indicated a high level of peptides at the interface have either hydrophobic residue L or I at the terminal position. There are no peptides in the 10 top that have charged groups present which is consistent with the previous observations. In the majority of cases, it appears that the best ranking peptides have a balance of hydrophobic groups, small residues and hydrophilic groups which is important for understanding how these peptides pack. The hydrophobic groups drive the peptide to the interface, the small groups allow for flexibility and rotational degrees of freedom which allows the peptide to orient itself, ensuring the hydrophilic groups interact with the aqueous layer.

**TABLE 2:** Highest and lowest ranking tetrapeptides based on %ADS.

10 Best %ADS		10 Worst %ADS	
Peptide	%ADS	Peptide	%ADS
PTAL	74.3	RRET	0.3
GAMI	72.3	RRES	0.5
AGGI	72.2	RRGH	0.7
AMSI	72.2	RRTN	0.8
AAMI	72.0	RRTD	0.8
LAAQ	71.3	RRDD	0.8
LAQG	70.7	ERRD	0.8
NLMH	70.7	SRRD	1.0
ANAL	70.3	QRRE	1.0
HGII	70.3	NRRD	1.0

On the other hand, the worst performing tetrapeptides contain the charged residues and hydrophilic residues. In these cases, the peptides will be able to interact with themselves and potentially aggregate, however, there is no hydrophobic residues to ensure that the peptide is driven towards the interface, as indicated by the substantially low %ADS values.

Based on the analysis of the initial screen we observed that the tetrapeptides had a lower number of candidates (12 *cf.* 32) that had a high adsorbance (>70%) at the interface. Therefore, we carried out extended timescale (10 µs) simulations of the best performing candidates, along with a selection of candidates with intermediate and low %ADS scores, and to determine whether the screening timescale affected the ability to identify good candidates. The selection includes those peptides with a %ADS (at 100 ns) that are: >60%, 1 at 50%, 1 at 40%, 1 at 30%, 1 at 20%, 1 at 10%, and 1 at 30%. These results (Table 3) indicate that the ability of the peptides to adsorb at the interface is increased as the simulation time is increased, although those that have a low adsorbance (<50% adsorbance after 100 ns) are unlikely to increase significantly given a longer simulation time. This suggests that the screening length is sufficient to help determine those candidates that can be excluded and also to help derive the design rules for creating tetrapeptides that have higher interfacial activity. However, it is also evident from the results that the %ADS after 100 ns cannot be exclusively used to rank the tetrapeptides after the 100 ns simulation, as the difference between the %ADS for high-scoring sequences decreases over longer timescales.

**TABLE 3:** Effect of simulation time on the %ADS for high scoring, intermediate, and low scoring tetrapeptides.

Peptide	%ADS (100 ns)	%ADS (10 µs)	Peptide	%ADS (100 ns)	%ADS (10 µs)
PTAL	74.3	100.0	LQCS	69.3	100.0
GAMI	72.3	99.7	LAGA	68.3	97.3
AGGI	72.2	100.0	AIAQ	67.0	92.7
AMSI	72.2	92.7	GLAG	64.7	94.5
AAMI	72.0	99.8	TAQL	64.7	98.0
LAAQ	71.3	91.2	GIAA	63.2	95.2
LAQG	70.7	90.0	LSQV	50.0	100.0
NLMH	70.7	99.3	AVGK	40.0	52.3
ANAL	70.3	88.2	ELNN	29.5	34.0
HGII	70.3	100.0	RRVE	20.0	4.0
AAAL	70.0	95.8	GNRR	10.0	5.3
IMLG	70.0	100.0	RRET	0.3	0.0

These extended systems can be further examined by looking at the density profiles of each of the systems to determine the ordering of the peptides at the interface (see Supplementary Material for details). The density profiles for these simulations demonstrate that, as expected, there is a high density of the hydrophobic residues positioned on the octane side of the interface, for the high-scoring candidates. Moreover, this method provides a good indication of the relative structuring of the peptides at the interface. The graphs can also be used in conjunction with the %ADS values to indicate which peptides form stable barriers. For example, the worst performing peptide RRET gives a low %ADS (0.00%) and with the density profile the graphs indicate poor structuring of the residues. On the other hand, looking at LQCS, which performs well, and it can be seen that the more hydrophobic groups L and C are situated at the oil phase whereas the hydrophilic groups Q and S are located at the aqueous phase. This would indicate that there is a high level of ordering and adsorption to the interface, which would support the formation of a stable emulsion.

Surface tension measurements, *i.e.*, studying the dynamic adsorption behavior of a selection of tetrapeptides to a water-air interface experimentally, were used to explore further the predictive ability of the simulations. Table 4 summarizes the five selected tetrapeptides and their characteristics (their primary amino acid sequence, molecular weight, and isoelectric point). All peptides, except for peptide RRET that is positively charged at neutral pH and being the most hydrophilic tetrapeptide, show no significant net charge (net charge ∼0).

**TABLE 4:** Experimentally characterized tetrapeptides selected from the simulation (see Table 3) via tensiometry.

Peptide	Molecular weight (Da)	pI^b^	Equilibrium surface tension (mN/m)	% ADS^a^ (100 ns)
PTAL	400.47	5.6	66.4	74.3
HGII	438.52	7.7	51.7	70.3
LQCS	449.52	5.1	60.3	69.3
LSQV	445.51	5.6	62.8	50
RRET	560.60	10.6	68.6	0.3

a Data from Table 3.

b Calculated using pepdraw.com at pH 7.

The general equilibrium adsorption behavior of the five tetrapeptides revealed significant differences. While the peptide RRET shows the highest equilibrium surface tension, γ(eq), of 68.6 mN/m, and the peptide HGII shows the lowest γ(eq) of 51.7 mN/m (at a concentration of 30 mM). This means that the latter is the most surface-active peptide followed by the peptide LQCS (with an γ(eq) of 60.3 mN/m); and peptide LSQV (γ(eq) of 62.8). Comparing this adsorption behavior with that of well-known surfactants, such as SDS, polysorbate *etc.*, we can conclude that the surface activity of the best peptide HGII is still significantly lower than that of commonly used surfactants (Santos et al., 2003; Kairaliyeva et al., 2017). For instance, the CMC (critical micellar concentration) of SDS is observed at a concentration of 8.2 mM inducing a minimal γ(eq) of 35 mN/m (pH 5.4, 25°C). However, the equilibrium surface tension of HGII (51.7 mM/m) is actually lower than that of β-casein (∼52 mM/m) or β-lactoglobulin (55 mN/m) at saturation surface coverage (Fainerman et al., 2020). The similarity in surface pressure between the tetrapeptides and intact proteins highlights the inherent efficacy of amino acid sequences at reducing interfacial tension.

**FIGURE 4:**
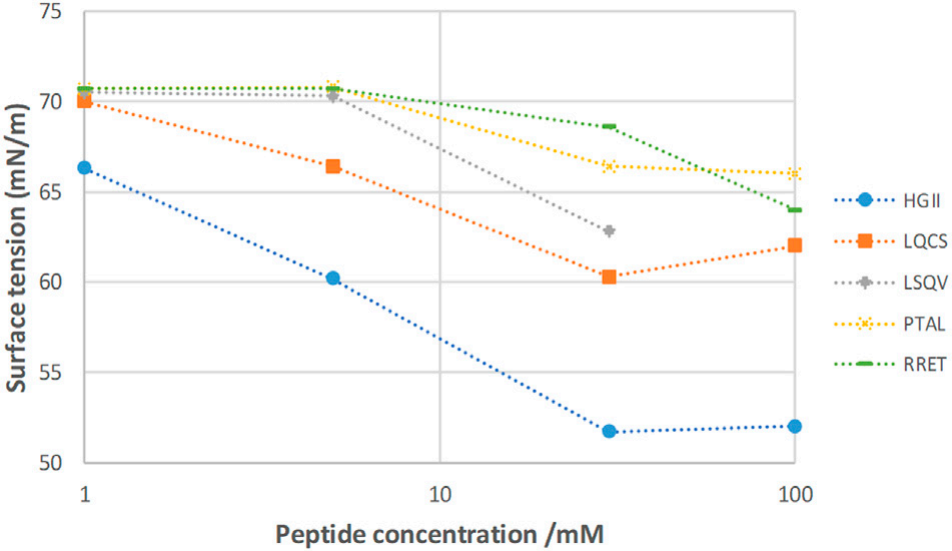
Adsorption isotherm of the five tetrapeptides; γ(eq) reached after 30 min of adsorption. HGII is the most surface-active and RRET the least surface-active peptide.

Figure 4 summarizes the equilibrium surface tension behavior of the five tetrapeptides as a function of molar concentration (adsorption isotherm). It confirms that peptide HGII is the most surface-active peptide indicating significant adsorption to the water-air interface. The lowest surface tension is attained at a concentration of 30 mM. The γ(eq) does not change anymore when increasing the peptide concentration up to 100 mM. This observation confirms that the HGII peptide shows a typical low molecular weight surfactant behavior, including the ability to self-assemble into, most probably, micelles at the so called “Critical Micellar Concentration”. A similar qualitative behavior is observed for the peptide LQCS.

The significant adsorption behavior of the HGII to a water-air interface is likely the result of its amphiphilic molecular structure, *i.e.*, having a polar and non-polar part. The hydrophilic amino acid Histidine is making up the polar head (p), and the more hydrophobic amino acids Glycine and Isoleucine the non-polar (n) end of the sequence. So, its structure can be simplified with p-n-n-n indicating amphiphilicity, representing a typical surfactant structure.

## Conclusion

Peptides have the ability to act as an emulsifier when they show a significant tendency to adsorb at interfaces. Therefore, the adsorption behaviour of peptides to an interface is a key parameter to evaluate in the screening process of best performing peptides in *e.g.*, emulsification. In this work it was shown that it is possible to design short (tetra) peptide-based emulsifiers that show good interfacial adsorption *in silico* and *in vitro*. The design process is achieved by applying coarse grain molecular dynamics simulation evaluating the % adsorbance (%ADS) of a certain, short-length peptide sequence. Initially, tripeptides were screened to evaluate whether particular peptide sequences give rise to higher adsorption abilities. Based on this analysis all potentially adsorbing tetrapeptide sequences were determined. Additionally, to verify the *in silico* results experimentally, five tetrapeptides (representing diverse amino acid sequences) were selected and synthesized: four that showed high %ADS; and one negative control.

The surface activity of these sequences was determined experimentally using tensiometry. The obtained results revealed good accordance between *in silico* and *in vitro* interfacial activity data and that the predicted sequence behavior performed similarly in experimentally determined aqueous-air interfaces. We thus confirmed tetrapeptides with high and low surface-activity behavior, as predicted computationally. However, the ranking of the well-adsorbing tetrapeptides was found to differ between *in silico* and *in vitro* results. This difference is likely due to the fact that the simulation work utilized a water-octane interface, while in tensiometry the adsorption to a water-air interface was studied. The latter experimental setup enables a robust surface-activity determination, while water-oil measurements bring about several difficulties, such as partial partitioning into the oil phase, and which needs to be compensated for. Nevertheless, this work presents for the first time that coarse-grain molecular dynamics simulations can be used to predict the sequence of tetrapeptides that feature surface-activity that then have been confirmed experimentally.

This computational screening approach offers the exploration of the vast amino acids sequence space now also for emulsifying tetrapeptides that, in contracts to conventional surfactants, bring about beneficial properties for their application in the cosmetic, and pharmaceutical industry. This approach does not have the ability to directly correlate with the experimental setup, or to reveal the detailed structure of the peptide conformations formed when creating these emulsions. However, it does provide an efficient and easily applicable approach for screening a large sequence space for a desired target property.

## Data Availability

Data has been made available in The Supplementary Material in the form of an Excel Spreadsheet. This data is also available from: https://doi.org/10.15129/c46efe5c-51ed-49fd-a53c-31df9fc8cc5f.
