# Supplementary material for: Directed Discovery of Tetrapeptide Emulsifiers
Source: Front Chem. 2022 Feb 17;10:822868. doi: 10.3389/fchem.2022.822868 (PMC8891517; doi:10.3389/fchem.2022.822868)
Supplement: Supplementary file 1 [file DataSheet1.PDF]

Supporting Information for Directed Discovery of Tetrapeptide Emulsifiers.

Gary G. Scott,[a] Tim Börner,[b] Martin E. Leser,[b] Tim J. Wooster,[b] Tell Tuttle[a*]

a. Department of Pure and Applied Chemistry, University of Strathclyde, 295 Cathedral Street, Glasgow, G1 1XL, UK

b. Institute of Materials Sciences, Nestlé Research Center, POB 44, CH-1000 Lausanne 26, Switzerland

Tripeptide Emulsion Investigation 2

Examination of the Tripeptide Systems at Longer Time Frames 4

Tetrapeptide Extended Timescale Investigation 5

Tripeptide Emulsion Investigation

All tripeptides without the amino acids F, W and Y were investigated. Each system was simulated for a period of 100ns before examination of the adsorption at the oil/water interface.

Table S1: Top ranked tripeptides (>70% %ADS)

Peptide	AP	%ADS	AP_{int-H}		Peptide	AP	%ADS	AP_{int-H}
LGA	1.33	83.00	0.28		GVG	1.24	71.00	0.25
GGL	1.35	76.00	0.28		LQA	1.36	71.00	0.19
IGA	1.34	75.67	0.25		NIA	1.38	71.00	0.19
LAG	1.21	74.33	0.21		PAS	1.45	70.83	0.25
AGP	1.29	74.17	0.29		SAP	1.42	70.83	0.24
GGI	1.35	74.17	0.28		ASI	1.52	70.83	0.20
PGG	1.32	73.83	0.34		AQL	1.36	70.83	0.18
ALA	1.29	73.50	0.18		PAT	1.39	70.67	0.23
AGI	1.41	73.33	0.25		AIA	1.30	70.67	0.18
IGQ	1.35	73.17	0.23		AVG	1.28	70.33	0.23
AIN	1.37	72.67	0.20		GIA	1.37	70.33	0.23
SAI	1.42	72.67	0.19		VAT	1.41	70.33	0.20
GIG	1.32	72.33	0.25		NQL	1.38	70.33	0.17
IAS	1.45	72.33	0.19		TAP	1.41	70.17	0.24
IGE	1.31	72.00	0.24		IQG	1.42	70.00	0.24
AGL	1.36	71.83	0.22		GLA	1.34	70.00	0.23

It is clear from the initial results that approximately a third of the tripeptides do not have any great affinity to adsorb to the interface (Figure S1). 1927 systems indicated adsorption of 50% or greater, with 32 systems showing a high level of adsorption (>70%, see Table S1).

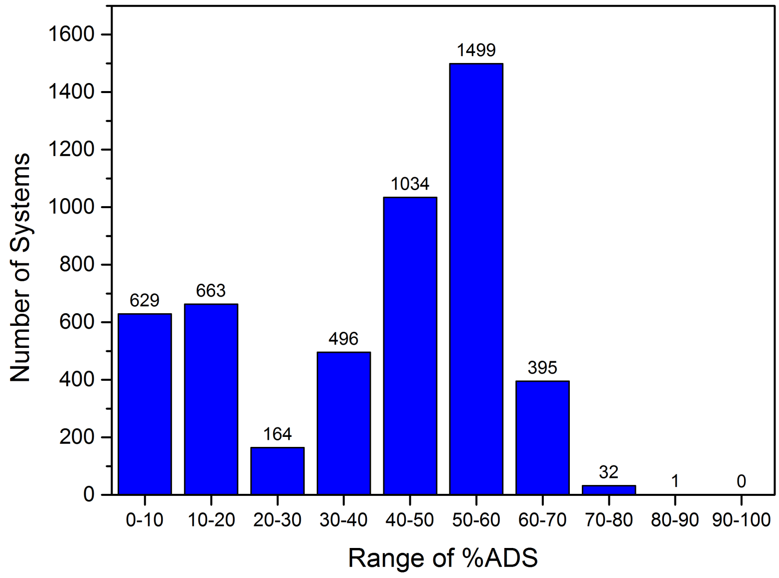

Figure S1: Number of systems adsorbing to the water/oil interface in bands of 10% ADS

From all the data collated from each system, important trends can be identified depending on the level of %ADS from every amino acid within each position on the tripeptide chain (Figure S2). It can be clearly observed that the presence of hydrophobic groups improve the peptides ability to adsorb to the water/oil interface. In all cases, the hydrophobic residues were found to prefer either terminal position, with a significant drop in %ADS observed when the hydrophobic group was located in the middle position. For hydrophilic amino acids, there was a significant drop in the average contribution from these amino acids on the %ADS. For the majority of amino acids, the middle position was the preferred position that showed improved adsorption.

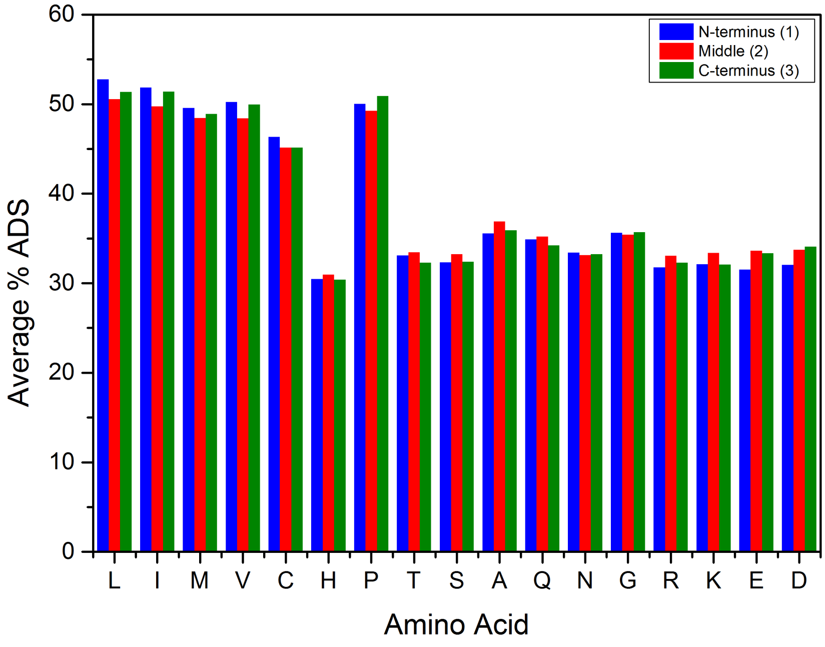

Figure S2: Resultant contribution of %ADS by each amino acid N-terminus (blue), middle (red) and C-terminus (green)

Examination of the Tripeptide Systems at Longer Time Frames

The top 32 tripeptides based on %ADS were simulated for a greater time frame to visualize the organisation of the peptides over a longer time frame. All the systems were subject to a 10 µs simulation outputting the data every 100 ns. This data was averaged and the standard deviation over the course of the simulation indicates the stability of the peptides at the interface. In all 32 cases the standard deviation was relatively low where the values ranged from 0.13 to 0.87 indicating that each system has stabilised (Table S2).

Table S2: Examination of the Stability of the Interfaces based on %ADS

Peptide	Average	Std. Dev.		Peptide	Average	Std. Dev.
GGI	99.21	0.25		ALA	95.43	0.57
ASI	98.82	0.23		TAP	95.41	0.13
IGA	98.78	0.31		AIG	95.23	0.33
LGA	98.78	0.33		AIA	95.18	0.53
GGL	98.77	0.31		GIA	95.12	0.34
AGI	98.73	0.26		GIG	95.11	0.31
PGG	98.61	0.46		IGQ	95.11	0.30
AGL	98.61	0.28		NIA	93.12	0.68
AGP	98.54	0.28		AIN	93.03	0.87
IAS	96.87	0.34		LQA	92.66	0.37
SAI	96.83	0.55		AQL	92.64	0.52
NGI	96.55	0.46		IQG	92.42	0.38
SAP	96.12	0.48		NQL	89.97	0.67
PAS	96.12	0.38		AVG	89.17	0.76
PAT	95.70	0.41		GVG	89.10	0.72
GLA	95.60	0.25		LAG	86.05	0.70

The top 32 peptides that showed high %ADS were simulated for a greater period of time (10 µs) and show increased average %ADS with low standard deviations. This indicates that the screening process successfully enables us to predict highly adsorbing tripeptides. Interestingly, peptides that contained glycine in the middle position showed a high level of ordering at the interface indicating that these peptides could be very good candidates for stabilising emulsions.

Tetrapeptide Extended Timescale Investigation

After the initial 100ns screening of the chosen tetrapeptides, a selection of tetrapeptides were chosen for extended analysis (100 ns to 10 us). 24 systems were chosen, the top ranking peptides which showed %ADS greater than 70% plus additional peptides which had ‘interesting’ sequences (Table S3).

Table S3: Results of tetrapeptides extended to 10us

Peptide	Log*P*	MW (g)	%ADS (100ns)	%ADS (10us)		Peptide	Log*P*	MW (g)	%ADS (100ns)	%ADS (10us)
PTAL	-0.36	400.46	74.33	100.00		LQCS	-0.04	449.51	69.33	100.00
GAMI	-0.14	390.49	72.33	99.67		LAGA	0.90	330.37	68.33	97.33
AGGI	1.68	316.35	72.16	100.00		AIAQ	0.65	401.45	67.00	92.67
AMSI	-0.83	420.51	72.16	92.67		GLAG	1.55	316.35	64.66	94.50
AAMI	-0.79	404.51	72.00	99.83		TAQL	0.27	431.48	64.66	98.00
LAAQ	0.52	401.45	71.33	91.17		GIAA	1.03	330.37	63.16	95.17
LAQG	1.17	387.43	70.66	90.00		LSQV	-0.48	445.51	50.00	100.00
NLMH	-0.96	513.61	70.66	99.33		AVGK	3.99	374.66	40.00	52.33
ANAL	0.60	387.42	70.33	88.17		ELNN	4.08	489.47	29.50	34.00
HGII	-0.98	438.53	70.33	100.00		RRVE	6.79	561.64	20.00	4.00
AAAL	0.25	344.39	70.00	95.83		GNRR	5.62	503.57	10.00	5.33
IMLG	-1.89	432.58	70.00	100.00		RRET	7.5	563.61	0.33	0.00

These extended systems can be further examined by looking at the density profiles of each of the systems to determine the ordering of the peptides at the interface (Figure S3). It would be expected to see that a high density of the hydrophobic groups would be seen at the oil side of the interface. This method gives a good representation of the structuring of the peptides at the interface. The graphs can also be used in conjunction with the %ADS values to indicate which peptides form stable barriers. For example, the worst performing peptide RRET gives a low %ADS (0.00%) and with the density profile the graphs indicate poor structuring of the residues. On the other hand, looking at LQCS, which preforms well, it can be seen that the more hydrophobic groups L and C are situated at the oil phase whereas the hydrophilic groups Q and S are located at the hydrophilic groups. This would indicate that there is a high level of ordering and adsorption to the interface therefore would form a strong emulsion.

AAAL

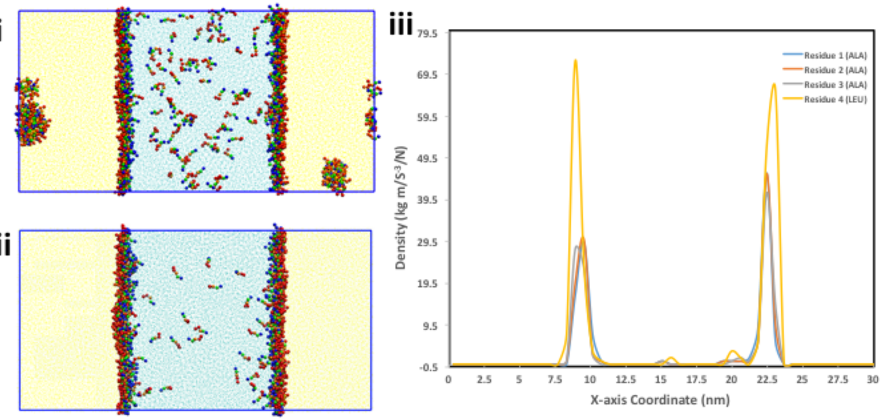

AAMI

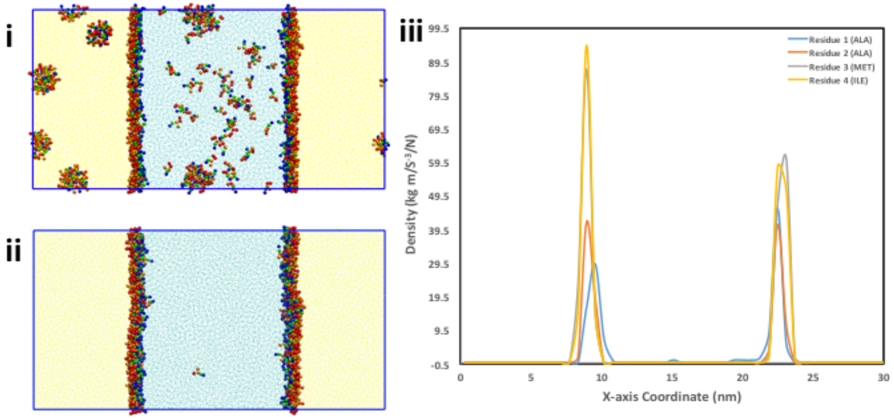

AGGI

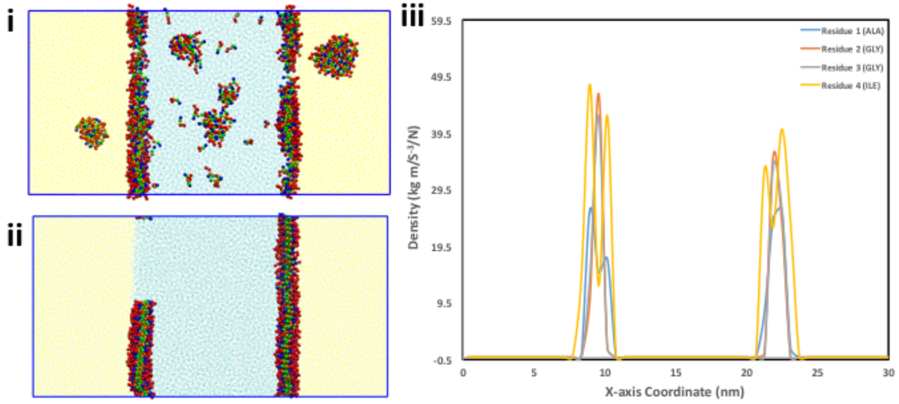

AIAQ

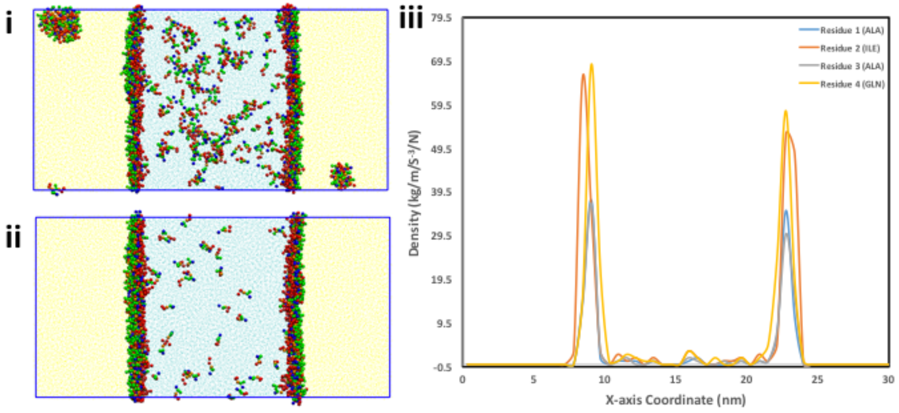

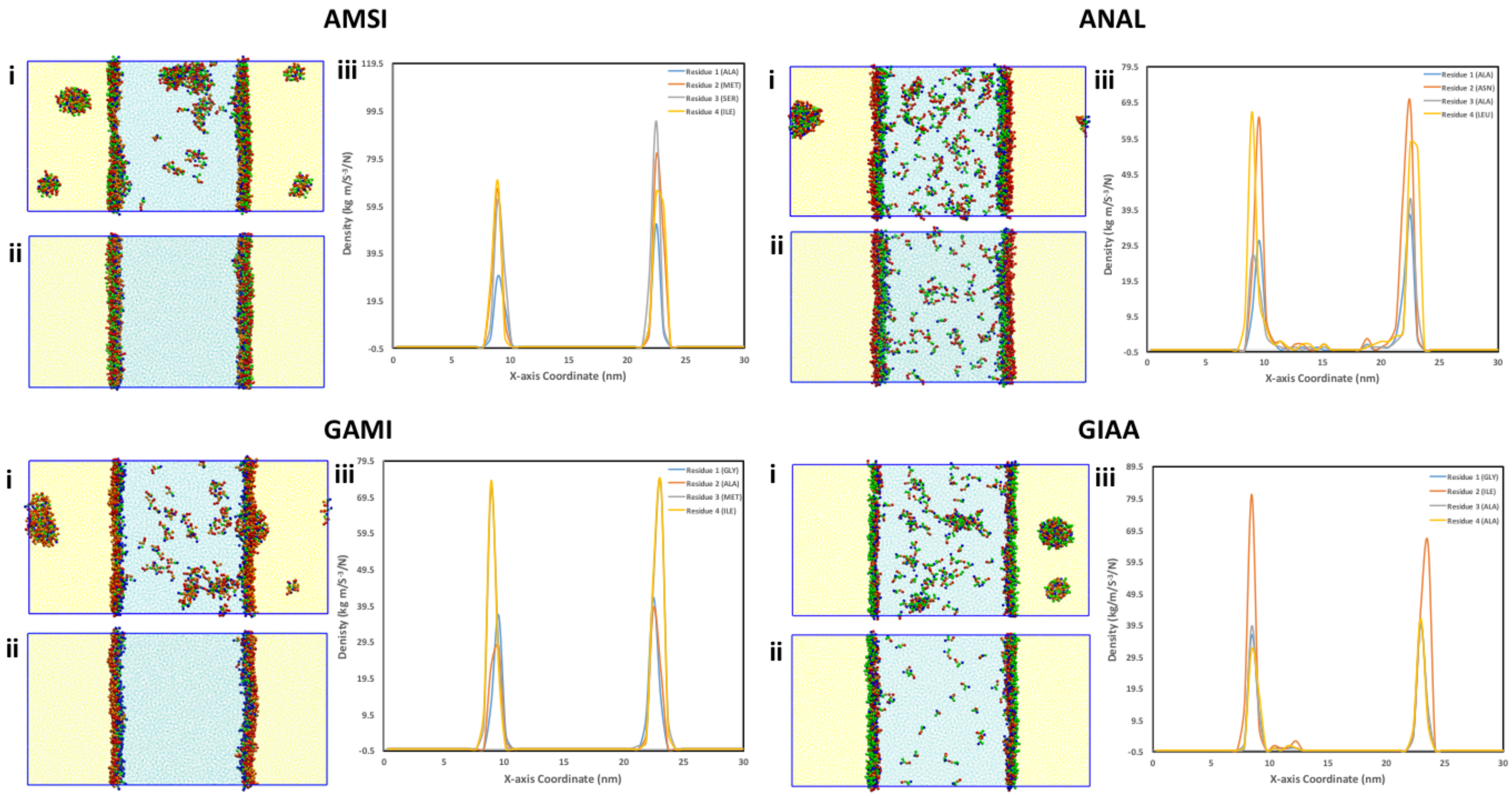
AMSI
ANAL
GAMI
GIAA
i
ii
iii
Residue 1 (ALA)
Residue 2 (MET)
Residue 3 (SER)
Residue 4 (ILE)
Residue 1 (ALA)
Residue 2 (ASN)
Residue 3 (ALA)
Residue 4 (LEU)
Residue 1 (GLY)
Residue 2 (ALA)
Residue 3 (MET)
Residue 4 (ILE)
Residue 1 (GLY)
Residue 2 (ILE)
Residue 3 (ALA)
Residue 4 (ALA)
X-axis Coordinate (nm)

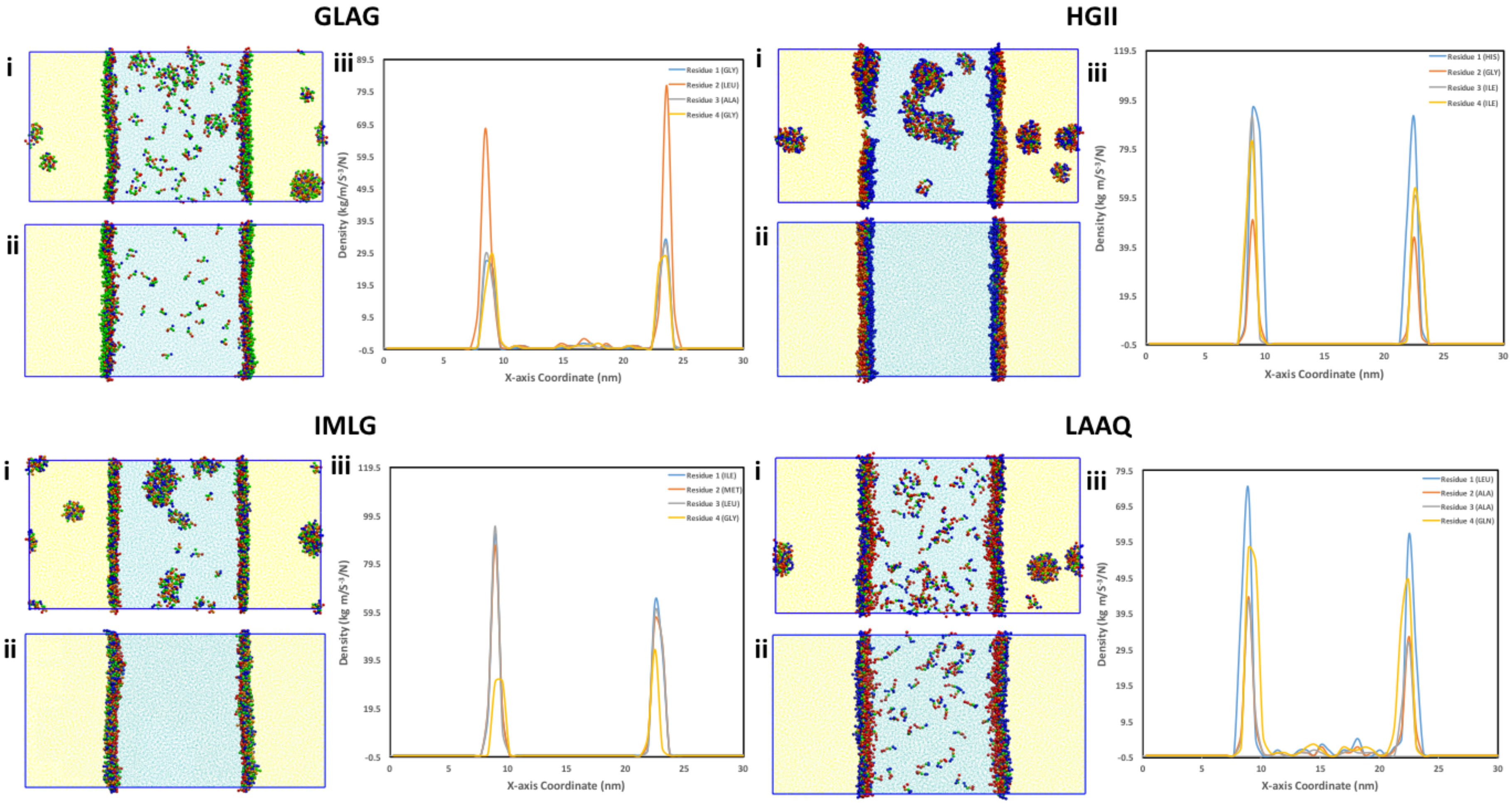
GLAG
i
ii
iii
Residue 1 (GLY)
Residue 2 (LEU)
Residue 3 (ALA)
Residue 4 (GLY)
Density (kg/m/S-3/N)
X-axis Coordinate (nm)
HGII
i
ii
iii
Residue 1 (HIS)
Residue 2 (GLY)
Residue 3 (ILE)
Residue 4 (ILE)
Density (kg m/S-3/N)
X-axis Coordinate (nm)
IMLG
i
ii
iii
Residue 1 (ILE)
Residue 2 (MET)
Residue 3 (LEU)
Residue 4 (GLY)
Density (kg m/S-3/N)
X-axis Coordinate (nm)
LAAQ
i
ii
iii
Residue 1 (LEU)
Residue 2 (ALA)
Residue 3 (ALA)
Residue 4 (GLN)
Density (kg m/S-3/N)
X-axis Coordinate (nm)

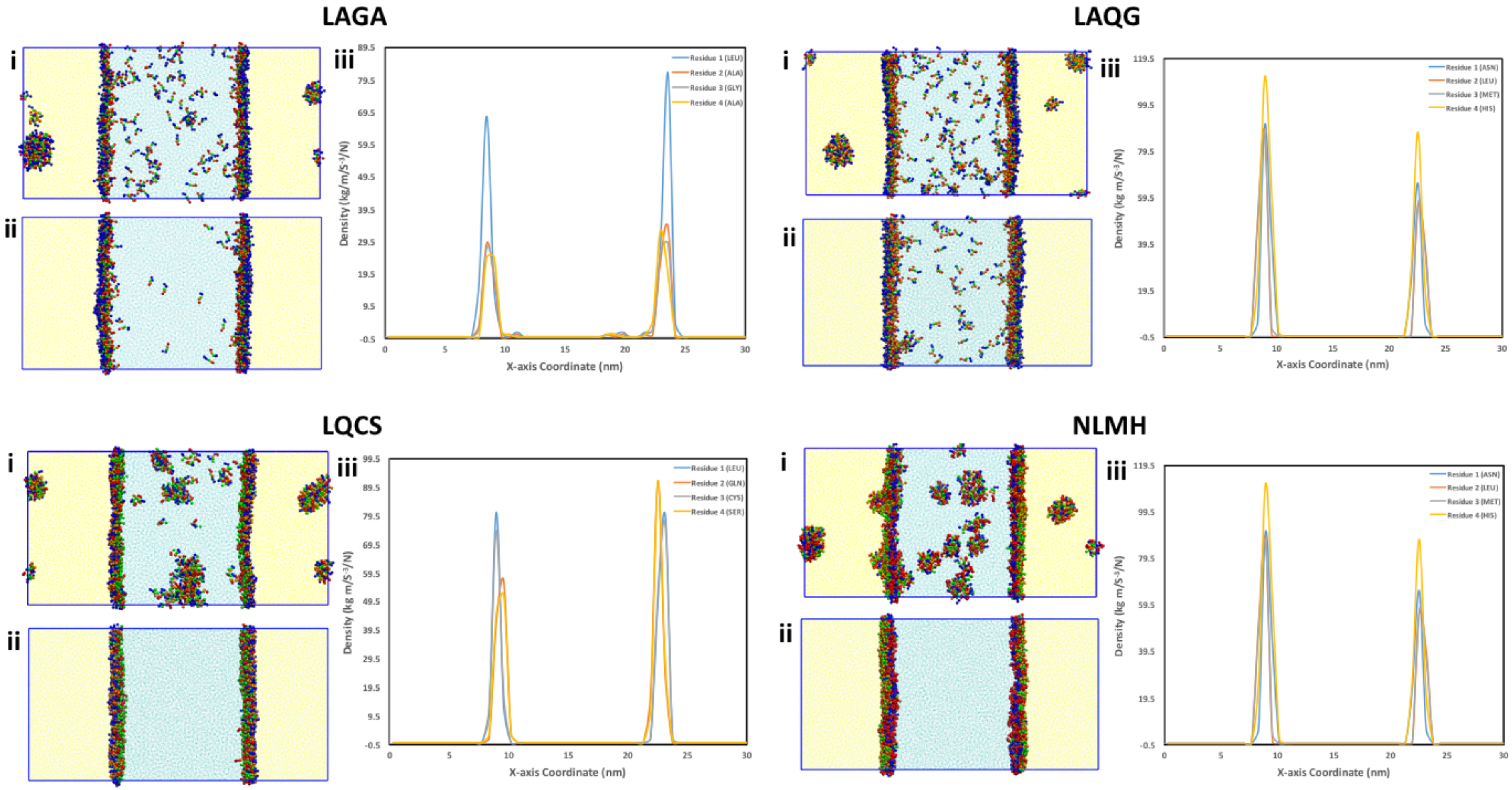
LAGA
i
ii
iii
Residue 1 (LEU)
Residue 2 (ALA)
Residue 3 (GLY)
Residue 4 (ALA)
Density (kg/m/S⁻³/N)
X-axis Coordinate (nm)
LAQG
i
ii
iii
Residue 1 (ASN)
Residue 2 (LEU)
Residue 3 (MET)
Residue 4 (HIS)
Density (kg m/S⁻³/N)
X-axis Coordinate (nm)
LQCS
i
ii
iii
Residue 1 (LEU)
Residue 2 (GLN)
Residue 3 (CYS)
Residue 4 (SER)
Density (kg m/S⁻³/N)
X-axis Coordinate (nm)
NLMH
i
ii
iii
Residue 1 (ASN)
Residue 2 (LEU)
Residue 3 (MET)
Residue 4 (HIS)
Density (kg m/S⁻³/N)
X-axis Coordinate (nm)

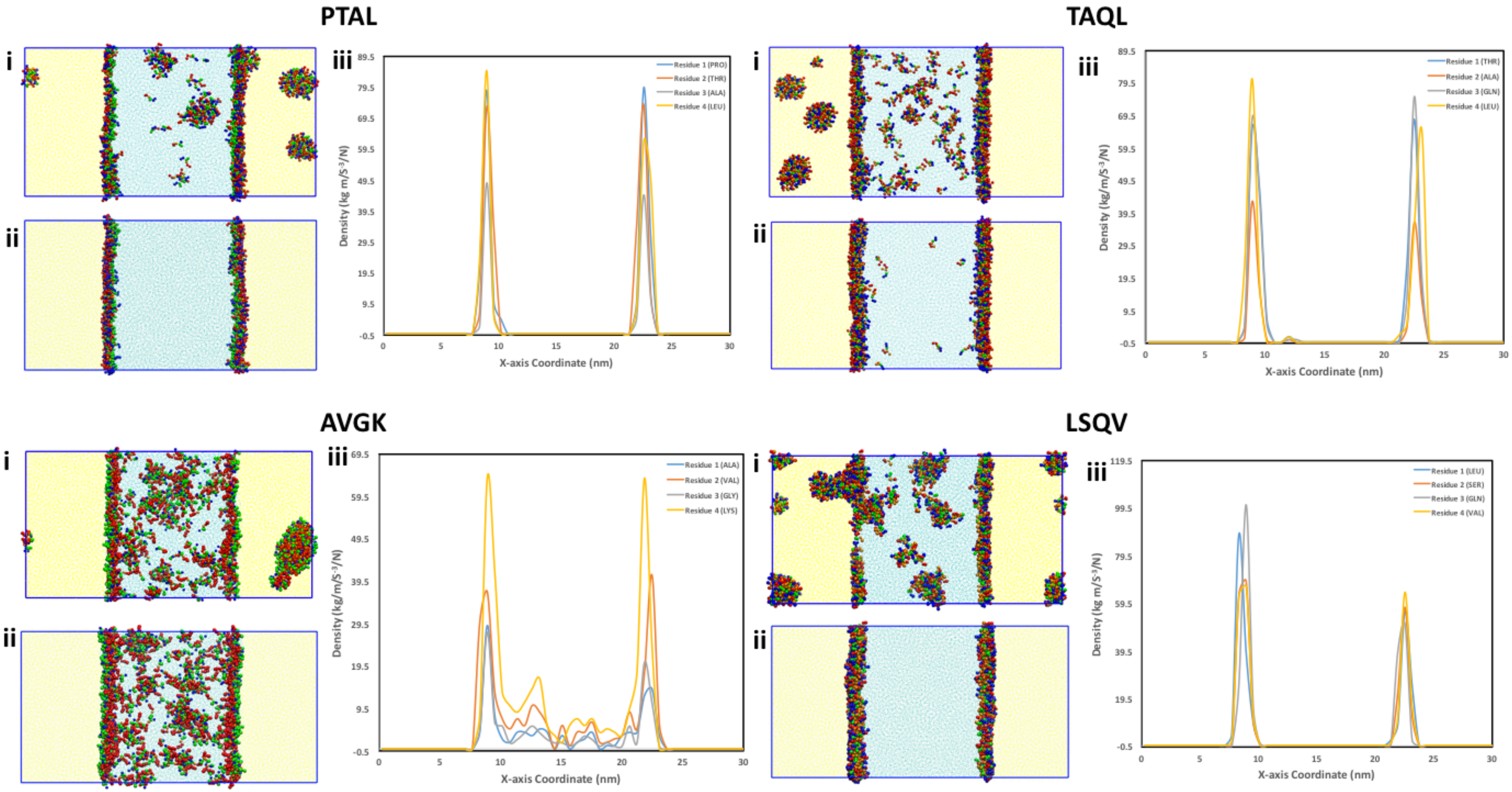
PTAL
i
ii
iii
Residue 1 (PRO)
Residue 2 (THR)
Residue 3 (ALA)
Residue 4 (LEU)
Density (kg m/S-3/N)
X-axis Coordinate (nm)
TAQL
i
ii
iii
Residue 1 (THR)
Residue 2 (ALA)
Residue 3 (GLN)
Residue 4 (LEU)
Density (kg/m/S-3/N)
X-axis Coordinate (nm)
AVGK
i
ii
iii
Residue 1 (ALA)
Residue 2 (VAL)
Residue 3 (GLY)
Residue 4 (LYS)
Density (kg/m/S-3/N)
X-axis Coordinate (nm)
LSQV
i
ii
iii
Residue 1 (LEU)
Residue 2 (SER)
Residue 3 (GLN)
Residue 4 (VAL)
Density (kg m/S-3/N)
X-axis Coordinate (nm)

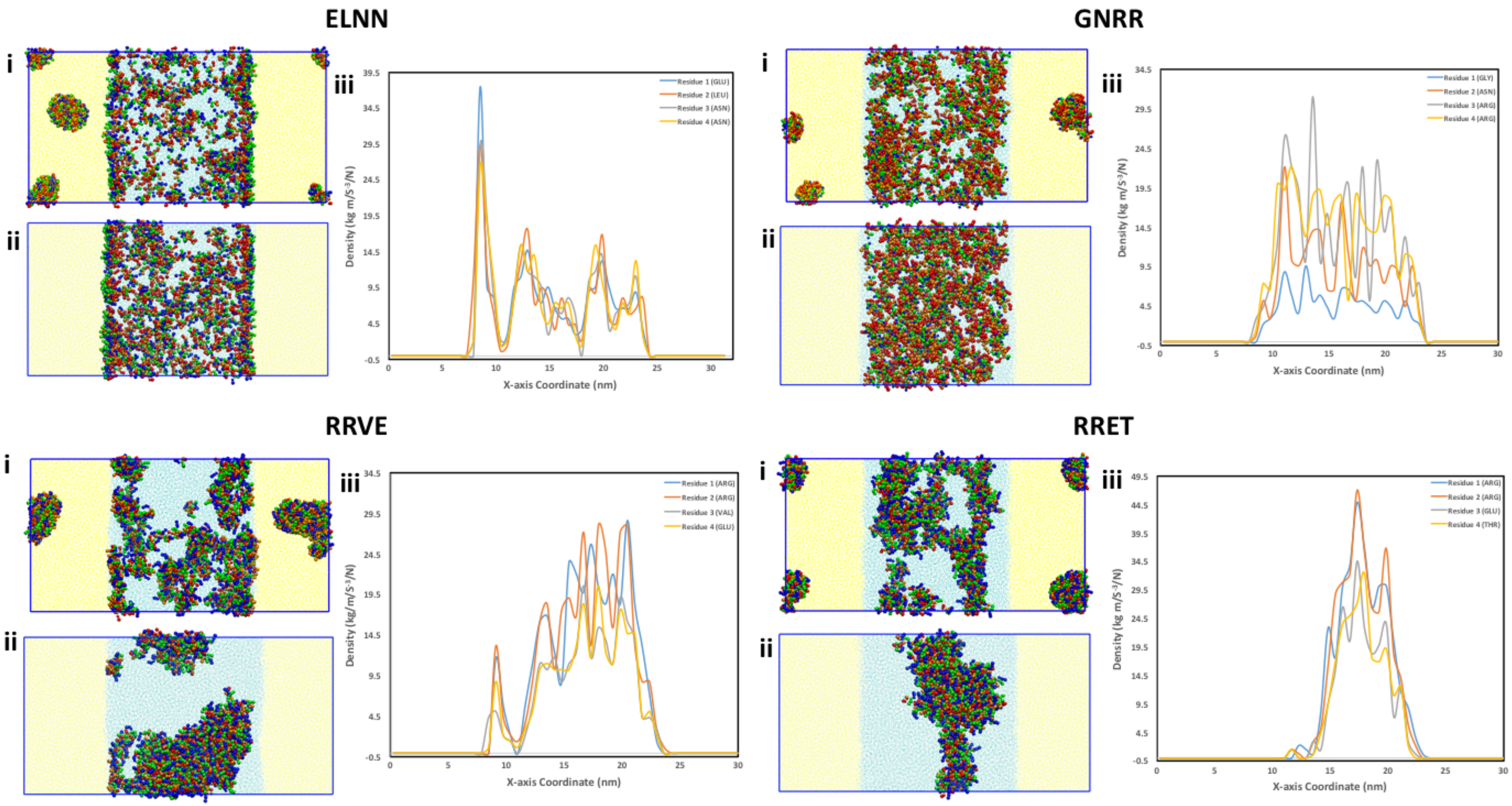

Figure S3: Extended simulations of 24 chosen systems. I)100ns ii) 10us iii) density profile for each residue at points along the x-axis (AA1 – Blue, AA2 – Green, AA3 – Orange, AA4 – Red
